# Adsorption and Gas Separation of Molecules by Carbon Nanohorns

**DOI:** 10.3390/molecules21050662

**Published:** 2016-05-19

**Authors:** Silvina M. Gatica, Anton Nekhai, Adam Scrivener

**Affiliations:** 1 Department of Physics and Astronomy, Howard University, 2355 Sixth Street NW, Washington, DC 20059, USA; 2 Rensselaer Polytechnic Institute, 110 Eighth Street, Troy, NY 12180, USA; nekhaa@rpi.edu; 3 Department of Computer Science, University of Rochester, 500 Joseph C. Wilson Boulevard, Rochester, NY 14627, USA; ascriven@u.rochester.edu

**Keywords:** adsorption, carbon nanohorns, carbon dioxide

## Abstract

In this paper, we report the results of Monte Carlo simulations of the adsorption of neon, argon, methane and carbon dioxide in carbon nanohorns. We model the nanohorns as an array of carbon cones and obtained adsorption isotherms and isosteric heats. The main sites of adsorption are inside the cones and in the interstices between three cones. We also calculated the selectivity of carbon dioxide/methane, finding that nanohorns are a suitable substrate for gas separation. Our simulations are compared to available experimental data.

## 1. Introduction

The investigation of adsorption phenomena has been a very exciting and successful scientific activity in the last half-century [1,2,3,4,5,6,7,8,9]. In recent years, a considerable amount of work has been devoted to investigate, theoretically and experimentally, the physical adsorption in bundles of carbon nanotubes and carbon nanohorns [10,11,12,13,14,15,16,17,18,19,20,21]. The separation of gas mixtures has become a widespread practice used in industry and society. Some examples of the applications of gas separation include: the purification of natural gas and air, the sequestration of CO, CO${}_{2}$ and other harmful gases and the production of H${}_{2}$. Within the last decade, environmental efforts have focused heavily on the reduction of CO${}_{2}$ in the atmosphere. The motive for targeting CO${}_{2}$ is a result of its “greenhouse gas effect” and its contribution to global warming.

Procedures for the separation of gases have also gained wide attention due to the importance that CO and H${}_{2}$ have managed to play in society. CO, for example, is a valuable material for the synthesis of a variety of chemicals. In many instances, synthesized CO contains several impurities, such as CO${}_{2}$, N${}_{2}$, H${}_{2}$, CH${}_{4}$ and H${}_{2}$O, which requires purification through gas separation methods. Furthermore, in the development of a hydrogen-based economy, the efficient production of H${}_{2}$ represents an issue for commercial applications. Regardless of the mechanism, H${}_{2}$ purification ultimately equates to a CO${}_{2}$ removal process. Within the arena of gas separation techniques, carbon nanohorns are a promising material.

“Physical adsorption” (physisorption) is a term applied to atoms or molecules that are weakly bound to surfaces. Physisorption has been extensively explored for more than half of a century because of interest in both potential applications and basic science. These applications include the separation of cryogenic gases, their storage and their use as surface characterization tools, such as the measurement of the surface area of porous media by nitrogen adsorption. The science of physisorption encompasses a wide variety of fundamental questions, including many related to phase transitions. Adsorption studies also serve a crucial purpose in fundamental and applied characterization of porous materials. In particular, nitrogen adsorption at 77 K is routinely used for the characterization of porous structure. In the case of substrates with pore dimensions <0.5 nm, activated diffusion may occur at 77 K [22], thereby preventing the observation of nitrogen uptake. In these circumstances, the adsorption of carbon dioxide at 273 K may be used as a routine measurement of micropore volume. Adsorption studies also increase the scientific community’s understanding of substrates when they are correlated with experimental results. The properties of adsorbates are thus one of the aspects of nanomaterials that needs to be assessed to enable its applications and to help bring its use in new technologies closer.

Gas separation by adsorption can be accomplished by three basic physical mechanisms: equilibria, kinetics and steric effects [23]. Equilibrium mechanisms rely on the strength of attraction between gas molecules and their substrate, while kinetic mechanisms involve the differences in the adsorption and transport rates of a gas on and through its substrate. Steric mechanisms, on the other hand, depend on the incompatibility between the size or shape of the adsorbate gas molecules and the pores of the substrate. For instance, since CO${}_{2}$ is typically found in a mixture with gases of similar size (for example, CH${}_{4}$ and H${}_{2}$), steric separation is not effective. However, CO${}_{2}$ possesses the strongest, attractive interactions with many substrates. For example, the energy of interaction of CO${}_{2}$ with graphene is twice as strong as CH${}_{4}$ with graphene [24]. As a result, the equilibrium mechanism presents the most plausible strategy.

Adsorption selectivity in a binary mixture of components *i* and *j* is defined as:

(1) $$S_{i/j}=\frac{x_{i}/x_{j}}{y_{i}/y_{j}}$$

where $x_{i}$ and $y_{i}$ are the molar concentrations of species *i* in the adsorbed phase and vapor phase, respectively. The concentration in the adsorbed phase, $x_{i}$, can be obtained from Monte Carlo or molecular dynamics simulations.

We can also estimate the selectivity of mixtures by the commonly-used “Ideal Adsorbed Solution Theory” (IAST) [25]. In the IAST, the selectivity is calculated using the data of the adsorption of the pure components, thus being a simple approach. Unfortunately, it may not be accurate for some mixtures that differ strongly in size or polarity. However, Babarao *et al*. confirmed that the theory is indeed reliable in the case of mixtures CO${}_{2}$ + CH${}_{4}$ in IRMOFs [26].

The specification of an appropriate adsorbent is the key for separation by adsorption. Although, currently, there are materials that are useful for gas separation, research in this area continues in order to optimize the performance of current materials and to examine a wider range of new sorbents. Many substrates have been used for this purpose, for example carbon nanotubes, activated carbons, zeolites and metallic organic frameworks.

Carbon nanohorns resemble short, wide, highly-defected single-walled nanotubes that end in a conical tip (“horns”) [27]. In contrast to regular nanotubes that assemble into parallel bundles, nanohorns form spherical aggregates with the nanohorns arranged along radial directions. The center of these aggregates is, in most cases, hollow. The body of the nanohorn is more or less cylindrical, with typical lengths between 30 and 70 nm and with a diameter that varies irregularly along its length (typical nanohorn diameters are between 3 and 8 nm). The diameters of these spherules are typically 80 to 100 nm.

Using the simulation technique grand canonical Monte Carlo (GCMC), we obtained the adsorption isotherms of Ne, Ar, CH${}_{4}$ and CO${}_{2}$ in an array of carbon nanohorns. We estimated the selectivity for CO${}_{2}$ based on the IAST approximation.

This article is organized as follows: In Section 2, we explain the method and the model. In Section 3, we describe our results, and in Section 4, we present our conclusions.

## 2. The Model

In the numerical study of adsorption, the following experiment is simulated: the substrate is exposed to a vapor at constant *P* and *T*. After some time passes, long enough to reach equilibrium, the uptake is measured under those *P* and *T* conditions. This can be simulated numerically by the methods of Grand Canonical Monte Carlo (GCMC) or Molecular Dynamics (MD) [28].

The GCMC method is based on statistical mechanics theory. The uptake is obtained at any given temperature and the chemical potential of the adsorbate. The chemical potential of the adsorbate is identical to that of the vapor, since both are in thermal equilibrium, and the last one is related to the pressure of the vapor, through the equation of state.

The inputs the data of the simulation are the pressure of the vapor, the temperature and the intermolecular forces. The output data of the simulation are the average number of adsorbed atoms (*N*), the averages of the total energy, the energy gas-surface ($E_{gs}$) and the energy gas-gas ($E_{gg}$). We also collect samples of the coordinates for each of the adsorbed atoms. For each single data point in the isotherms N(P,T), we typically run $3\times 10^{6}$ MC moves to reach equilibrium, and $10^{6}$ moves are performed for data collection. The ratio of creation/deletion/translation moves is 0.40/0.40/0.20. For details on the method, we refer the reader to [18].

**Figure 1:**
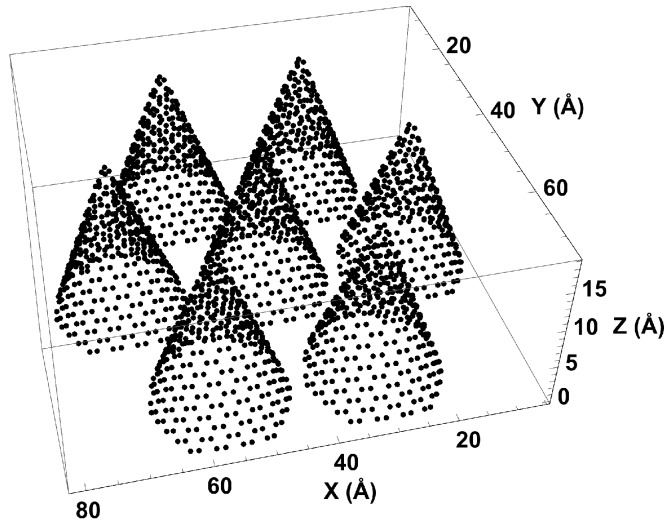
Simulation cell with seven nanohorns.

Carbon nanohorns have two main sites of adsorption: inside the cones near the tip and in the external interstices between cones. We designed a simple version of the nanohorns, consisting of an array of seven “graphene” cones on the 8.0 nm × 8.7 nm base of the simulation cell (see Figure 1). The cones are identical, with 2 nm-diameter bases and 1.5-nm heights. Although the model lacks the spherical curvature typical of a dahlia-shaped arrangement, it holds the main features that are important for adsorption: interstices and conical tip. The boundary conditions are set reflective in the *z* direction and periodic in x,y.

We compute the adsorption potential as the pairwise sum of two-body interactions between individual carbon atoms and the adatom,

(2) $$V\left(\vec{r}\right)=\sum_{i}U(|\vec{r}-{\vec{R}}_{i}\left|\right)$$

For Ar, Ne and CH${}_{4}$, we use the Lennard–Jones (LJ) potential to model the adsorbate-adsorbate and adsorbate-carbon interactions.

The CO${}_{2}$ molecule was represented as a rigid three-site linear molecule with partial charges in each site [29,30], $q_{O}=-288e$ and $q_{C}=+0.576e$ and a bond length of 1.18 Å. These parameters were set to mimic the molecular quadrupole moment, Q=−4.3 B. The interaction energy between a CO${}_{2}$ molecule and each carbon atom in the nanohorn is the sum of LJ terms:

(3) $$U_{CO2-C}=\sum_{i}U_{LJ}^{iC}\left(r_{iC}\right)$$

while the CO${}_{2}$-CO${}_{2}$ interaction is obtained as a sum of the LJ and electrostatic Coulomb terms:

(4) $$U_{CO2-CO2}=\sum_{ij}\left(U_{LJ}^{ij}\left(r_{ij}\right)+k_{e}\frac{q_{i}q_{j}}{r_{ij}}\right)$$

These sums are done over the carbon and oxygen atoms in the CO${}_{2}$ molecule.

The LJ parameters for the adatom-C potential are obtained by fitting the physical properties of the gases and using semi-empirical combining rules: $\sigma_{aC}=\left(\sigma_{aa}+\sigma_{CC}\right)/2$ and $\epsilon_{aC}=\sqrt{\epsilon_{aa}\epsilon_{CC}}$ [31], with $\sigma_{CC}=3.4$ Å and $\epsilon_{CC}=28$ K [17,32].

The LJ parameters are exhibit in Table 1.

## 3. Results

The simulations were done for Ne, Ar, CO${}_{2}$ and CH${}_{4}$. The results are described below.

**Figure 2:**
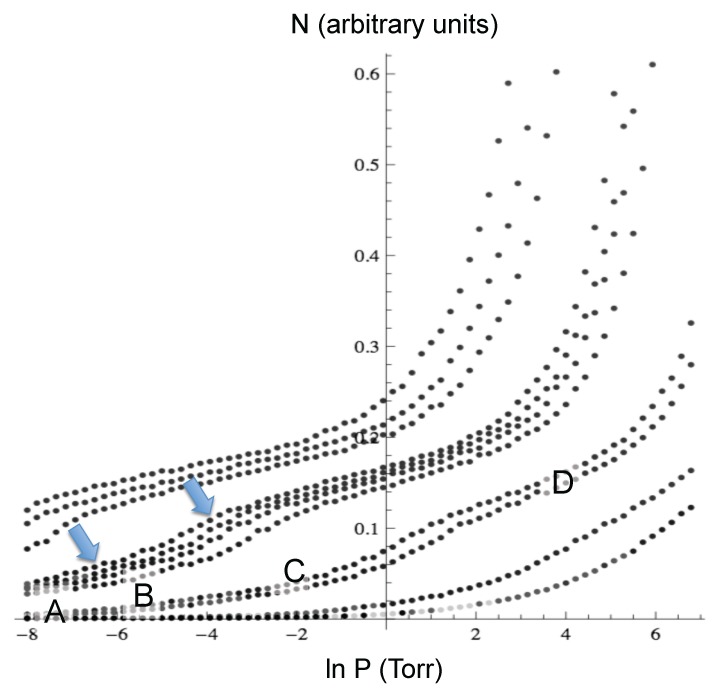
Adsorption isotherms for Ne at temperatures between 18.7 K and 49.8 K. The arrows indicate the bumps, and the labels A to D correspond to the configurations shown in Figure 3.

**Figure 3:**
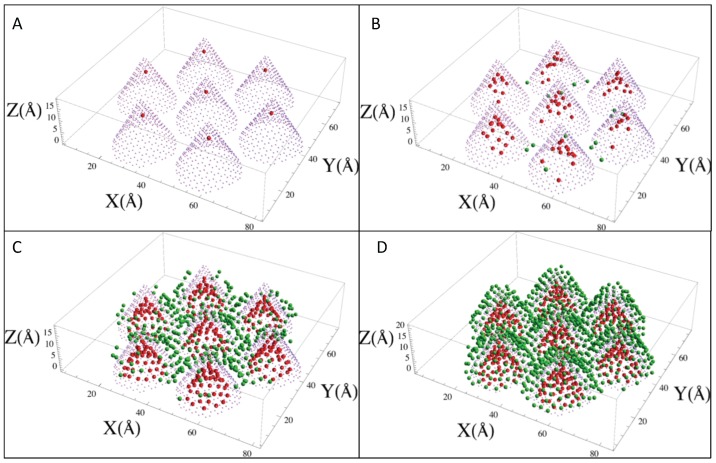
Sample configurations (at T, P conditions, indicated by the labels **A** to **D** in Figure 2 showing Ne atoms inside the cones (red) or outside (green).

Neon: Figure 2 shows the adsorption isotherms for Ne at temperatures between 18.7 K and 49.8 K, and Figure 3 shows the neon atoms in the simulation cell at four different P,T conditions as labeled A to D in Figure 2. At the lowest coverage, the neon atoms are adsorbed in the most attractive sites, which are inside the cones (labeled A in the isotherms). At higher pressure, the adsorption occurs both inside and in the external interstices (label B). Adsorption continues to increase until the least attractive sites are populated, which are the walls of the cones opposite the interstices (label C), and finally, a “monolayer” forms covering all of the exterior of the cones (label D). The isotherms show a clear “bump” between C and D, similar to a monolayer completion usually seen in adsorption experiments, and another almost imperceptible bump at low coverage. The experiments reported in [19] show two bumps, as well. The pressure at which the condensation starts in the simulations is similar to the one in the experiment at the same temperature. However, in the simulations, the condensation step is less abrupt.

The quantum effects on the Ne isotherms are small. We conclude this after calculating the upper limit to both the Boer parameter Γ and the zero-point energy (ZPE). Those correspond to the “worst-case scenario” in our simulations, that is T=18.7 K and Ne-Ne separation $=\sigma_{LJ}$, in which case, we obtain Γ=0.3 and ZPE =0.1 K (much smaller than the thermal, Ne-Ne and Ne-graphene energies).

We computed the isosteric heat of adsorption from the isotherms, to compare to the experimental results from [19].

**Figure 4:**
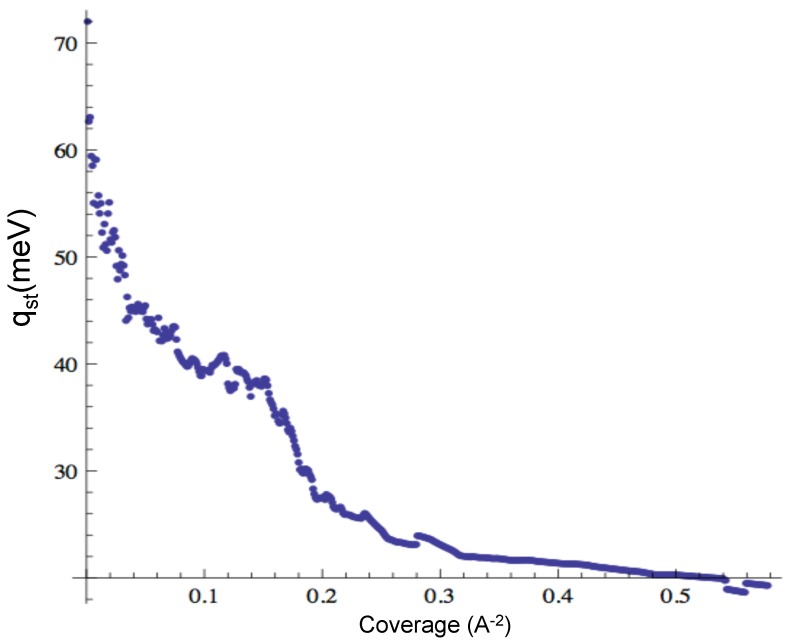
Isosteric heat of adsorption of Ne.

The isosteric heat of adsorption, $q_{st}$, is defined as:

(5) $$q_{st}\left(n,T\right)=k_{B}T^{2}{\left(\frac{\partial \ln P}{\partial T}\right)}_{n}$$

where *n* is the coverage and *P* is the vapor pressure at the given n,T. We compute the $q_{st}$ from the adsorption isotherms at a constant value of *n*. The result is displayed in Figure 4, which can be compared to the experimental result from [19].

We observe a plateau at approximately 20 meV, similar to the experimental value (21 meV). However, the peak value in our simulations is higher than in the experiment (37.5 meV). This indicates an overestimation of the adsorption energy inside the cones, probably an effect of the “pointy shape” of the cones.

**Figure 5:**
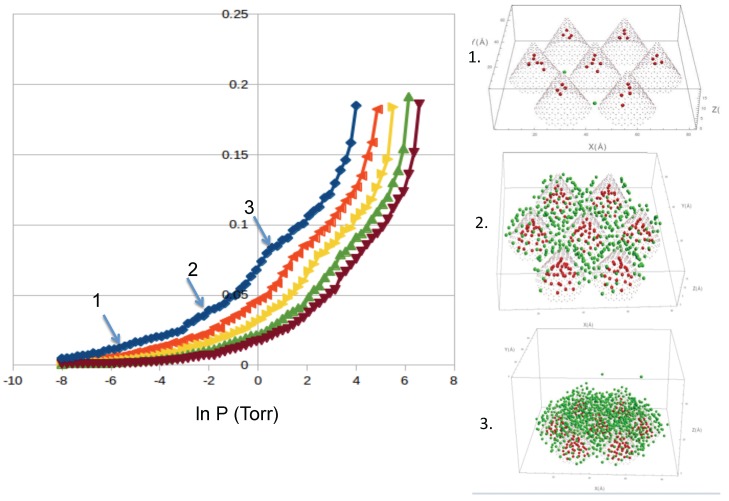
Adsorption isotherms of argon (in Å${}^{-2}$), at temperatures of 70.32, 81.51, 86.45 and 91.13 K (from left to right). Also shown are configurations at low (**1**); medium (**2**) and high (**3**) coverage, as labeled. Color code: atoms inside the cones (red) and outside (green).

**Figure 6:**
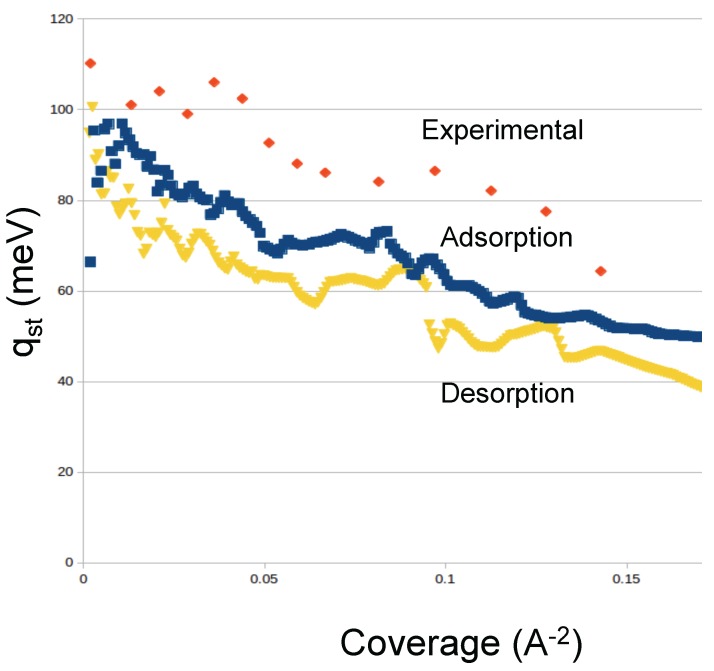
Isosteric heat of adsorption and desorption of argon. Also shown are the experimental data from [20].

Argon: We simulated the adsorption of Argon at temperatures from 70 to 91 K in order to be able to compare to the available experimental data. Our results for Ar show similar features as Ne and reasonable agreement with the experimental results from Calvillo *et al.* [20]. We show the results in Figure 5. We identify two “bumps” at low coverage and medium coverage. In our simulations, the first bump corresponds to the occupancy of the interstices, and the second bump to the complete coverage of the external sites. We also see that the condensation in the cell starts at similar pressures as in the experiment [20]. The desorption isotherms show a slight hysteresis in the condensation regime. We also computed the isosteric heat of adsorption, shown in Figure 6. We find that the values of $q_{st}$ obtained in our simulations for both adsorption and desorption are lower than the experimental q${}_{st}$ [20]. We believe that the reason for the discrepancy is the simplicity of our nanohorns.

**Figure 7:**
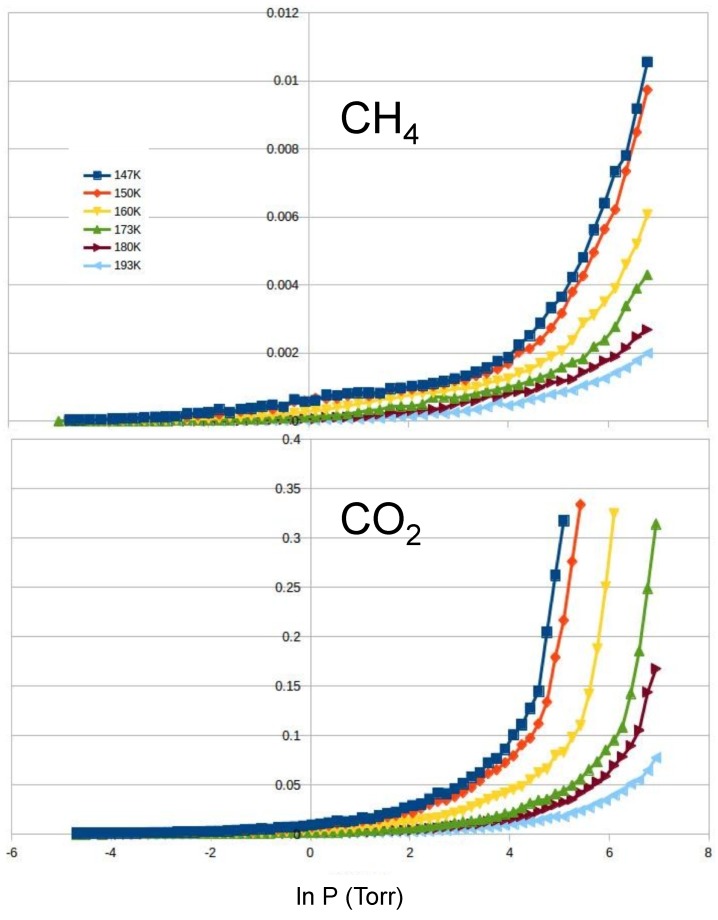
Adsorption isotherms (in arbitrary units) of methane (**top**) and carbon dioxide (**bottom**) at temperatures of 147, 150, 160, 173, 180 and 193 K from left to right.

CO${}_{2}$ and CH${}_{4}$: In order to address the selectivity of mixtures, we run simulations for pure carbon dioxide and pure methane at temperatures from 143 to 193 K (see Figure 7). We found that CO${}_{2}$ is adsorbed at significantly lower pressure than CH${}_{4}$. As a result, the substrate adsorbs CO${}_{2}$ with high selectivity. From our isotherms and using the IAST approximation, we estimate the values of $S_{CO2/CH4}$: 25, 23, 16, 10, 8 and 6, for temperatures of 143 K, 150 K, 160 K, 173 K, 180 K and 193 K, respectively.

**Figure 8:**
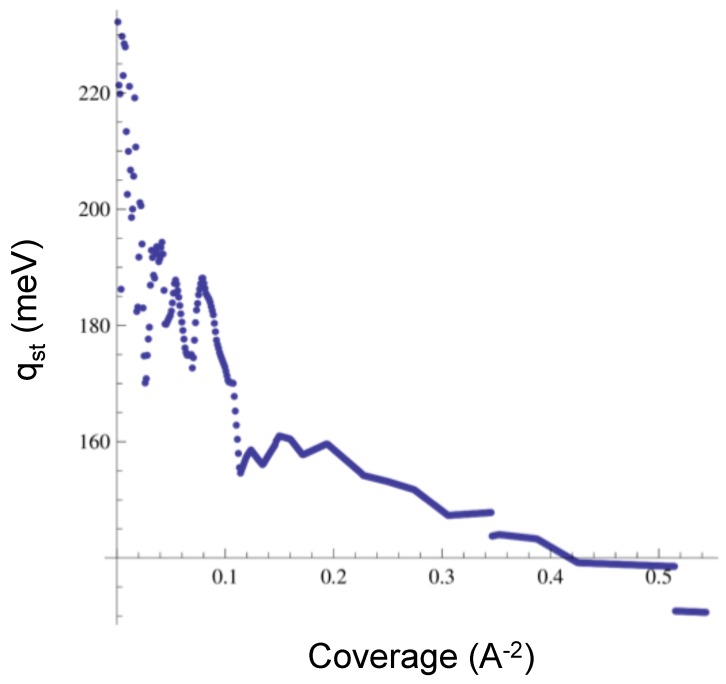
Isosteric heat of adsorption for CO${}_{2}$.

We also obtained the isosteric heat of adsorption for CO${}_{2}$, which we display in Figure 8 for low to medium coverage. The low coverage limit is 230 meV, and the medium coverage value is near 140 meV. These values are lower than the experimental data, which range from 237 to 280 meV [21]. However, we find agreement with the experiment in the fact that the isotherms for CO${}_{2}$ are smooth (no sub-steps or bumps), unlike the isotherms for Ar or Ne. The isosteric heat does not show a “u” shape like the experimental $q_{st}$ reported in [21]. In order to address this feature, we should obtain the values of the $q_{st}$ at higher coverage, a regime in which we have no results to report yet.

## 4. Conclusions

We have simulated the adsorption of neon, argon, carbon dioxide and methane in a simplified version of carbon nanohorns. Given the approximations made in our model, we have found close agreement with the experimental data available. The main simplification of our model is that there are only seven identical cones arranged on a flat lattice repeated periodically, while the real substrate is a spherical array of cones with different sizes and aspects.

The coincidences with the experimental data are many. For example, the isotherms of neon and argon feature bumps similar to those in the real substrate. Hence, we can conclude that those bumps observed in the experiment correspond to adsorption in the internal tip and interstices. The isotherms of carbon dioxide, on the contrary, are smooth, implying that internal and interstitial sites may have similar energies. The quantitative agreement is better for neon and argon than for CO${}_{2}$. One possible reason is that the adsorption of Ne and Ar is highly dominated by the two first sites, while for CO${}_{2}$, the external surface may be more significant. One of the simplifications of our model is that the substrate has only seven cones with three interstices; hence, the “exterior” is overexposed.

We also estimated, using the IAST, a high selectivity of CO${}_{2}$/CH${}_{4}$. A more precise calculation would require a heavier computation, either MC or MD of the CO${}_{2}$ + CH${}_{4}$ mixture on a more realistic substrate. Our results can be used as a guide to the conditions of temperature and vapor pressure in which those calculations would be worth it.

## Figures and Tables

**Table 1:** Lennard–Jones (LJ) parameters used in the simulations.

Adatom	σ (Å)	ϵ (K)
Ar [33]	3.4	120
Ne [33]	2.75	35.6
CH${}_{4}$ [34]	3.7	148
C in CO${}_{2}$ [29,30]	2.8	29.7
O in CO${}_{2}$ [29,30]	3.0	83
